# Selective transduction of cerebellar Purkinje and granule neurons using delivery of AAV-PHP.eB and AAVrh10 vectors at axonal terminal locations

**DOI:** 10.3389/fnmol.2022.947490

**Published:** 2022-09-13

**Authors:** Magdalena Surdyka, Ewelina Jesion, Anna Niewiadomska-Cimicka, Yvon Trottier, Żaneta Kalinowska-Pośka, Maciej Figiel

**Affiliations:** ^1^Department of Molecular Neurobiology, Institute of Bioorganic Chemistry Polish Academy of Sciences, Poznań, Poland; ^2^Institute of Genetics and Molecular and Cellular Biology, INSERM U1258, CNRS UMR7104, University of Strasbourg, Illkirch, France

**Keywords:** adeno-associated virus (AAV), AAVrh10, AAV-PHP.eB, cerebellum, deep cerebellar nuclei, Purkinje cells, granule cells, retrograde

## Abstract

Adeno-associated virus (AAV)-based brain gene therapies require precision without off-targeting of unaffected neurons to avoid side effects. The cerebellum and its cell populations, including granule and Purkinje cells, are vulnerable to neurodegeneration; hence, conditions to deliver the therapy to specific cell populations selectively remain challenging. We have investigated a system consisting of the AAV serotypes, targeted injections, and transduction modes (direct or retrograde) for targeted delivery of AAV to cerebellar cell populations. We selected the AAV-PHP.eB and AAVrh10 serotypes valued for their retrograde features, and we thoroughly examined their cerebellar transduction pattern when injected into lobules and deep cerebellar nuclei. We found that AAVrh10 is suitable for the transduction of neurons in the mode highly dependent on placing the virus at axonal terminals. The strategy secures selective transduction for granule cells. The AAV-PHP.eB can transduce Purkinje cells and is very selective for the cell type when injected into the DCN at axonal PC terminals. Therefore, both serotypes can be used in a retrograde mode for selective transduction of major neuronal types in the cerebellum. Moreover, our *in vivo* transduction strategies are suitable for pre-clinical protocol development for gene delivery to granule cells by AAVrh10 and Purkinje cells by AAV-PHPeB.

## Introduction

Currently, the most promising viral vectors to deliver genetic therapeutics in the brain are based on adeno-associated viruses (AAVs). AAV vectors are already used clinically as a component of approved gene therapeutics and in a rapidly increasing number of therapeutic trials in central nervous system (CNS) disorders (Kuzmin et al., 2021; Research CBER, 2021). The clinical use demonstrated that AAV vectors are safe, well-tolerated, show relatively low immunogenicity, demonstrate high transduction efficacy, and capacity to transduce dividing and quiescent cells *in vivo* (Hadaczek et al., 2010; Nathwani et al., 2011; Choudhury et al., 2017). The AAVs show cell-type specificity determined by viral capsid proteins recapitulating different serotypes. The most frequently studied serotypes of AAV vectors are 1, 2, 5, 8, 9, and recombinant human (rh)10 (Cearley and Wolfe, 2006; Klein et al., 2008; Chan et al., 2017). However, the major challenge in brain AAV-based therapies is targeting specific cell populations and brain regions for selective delivery of therapeutics exclusively to the diseased neural cells.

The cerebellum contains clinically essential cell types in many neurodegenerative disorders (Cendelin, 2014; Klockgether et al., 2019; Franklin et al., 2021). Moreover, the cerebellar neuronal network is a model of neuronal connectivity in the brain (De Zeeuw et al., 2021). The cytoarchitecture of the cerebellum and its neuronal connections are relatively well-described, particularly in model animals (Leto et al., 2016). In brief, the cerebellum consists of several layers and structures, such as the molecular layer (ML) containing the Purkinje cell (PC) dendritic tree and stellate neurons and the Purkinje cell layer (PCL) containing PC bodies. PCL and ML also contain GFAP-positive glial cells called Bergmann glia (Goertzen and Veh, 2018; Wizeman et al., 2019). Another cell type located close to the PC somatodendritic compartment is called basket neuron, which has processes located tightly around PC bodies. Such tight pericellular structure of processes can be detected by immunostaining and is often referred as pinceau. Moreover, the granule cell layer (GCL) is located internally from ML and PCL, and contains the bodies of granule cells (GC), while their axons project toward outer cerebellar layers, split (bifurcate), and run in ML as parallel fibers. The most internal part of the cerebellum is the cerebellar white matter (WM), which contains deep cerebellar nuclei (DCN). All neurons of the cerebellum build complex neuronal networks. For instance, the split axons of granule cells in the ML make synapses with the PC dendrites. The WM contains a dense fiber tract network of PC axons that terminate at neurons located in DCNs (Gill and Sillitoe, 2019). Based on the location of the axonal terminals, there are “hotspots” in the cerebellar cytoarchitecture that are suitable for AAV administration to induce the retrograde transduction of selected cell populations.

The cerebellum controls muscle coordination, equilibrium, and posture, relying on various cell types and connections between these cells (Manto et al., 2012; Perciavalle et al., 2013; Lang et al., 2017). Therefore, the targeted and effective delivery of AAV-based therapeutics to the cerebellar subregions and cell populations is essential for the therapeutic outcome in neurodegenerative disorders.

Therefore, the work aimed to find the most suitable cerebellar AAV serotypes and combine them with appropriate delivery (local and systemic) and transduction modes (direct or retrograde) to target specific cell populations in the mouse cerebellum. We have selected AAVrh10 and AAV-PHP.eB serotypes for further studies and present effective and selective transduction strategies for the Purkinje and granule neurons. Importantly, the strategy makes use of cerebellar connectivity and placing of AAV at axonal terminals (retrograde transduction). In addition to main cell types, such as PC and GC, we also demonstrate the conditions for transduction of other cerebellar cell types. Our work paves the way for a better understanding of possible outcomes of AAV injections, which are influenced by serotype, delivery mode, and region of injection. Our results contribute to the design of improved cell-specific delivery systems for (pre)clinical gene therapies.

## Results

### Early AAVrh10_eGFP transduction into lobules produces eGFP+ cells in cerebellar ML with hardly any GFP signal in Purkinje cells

**Figure 1:**
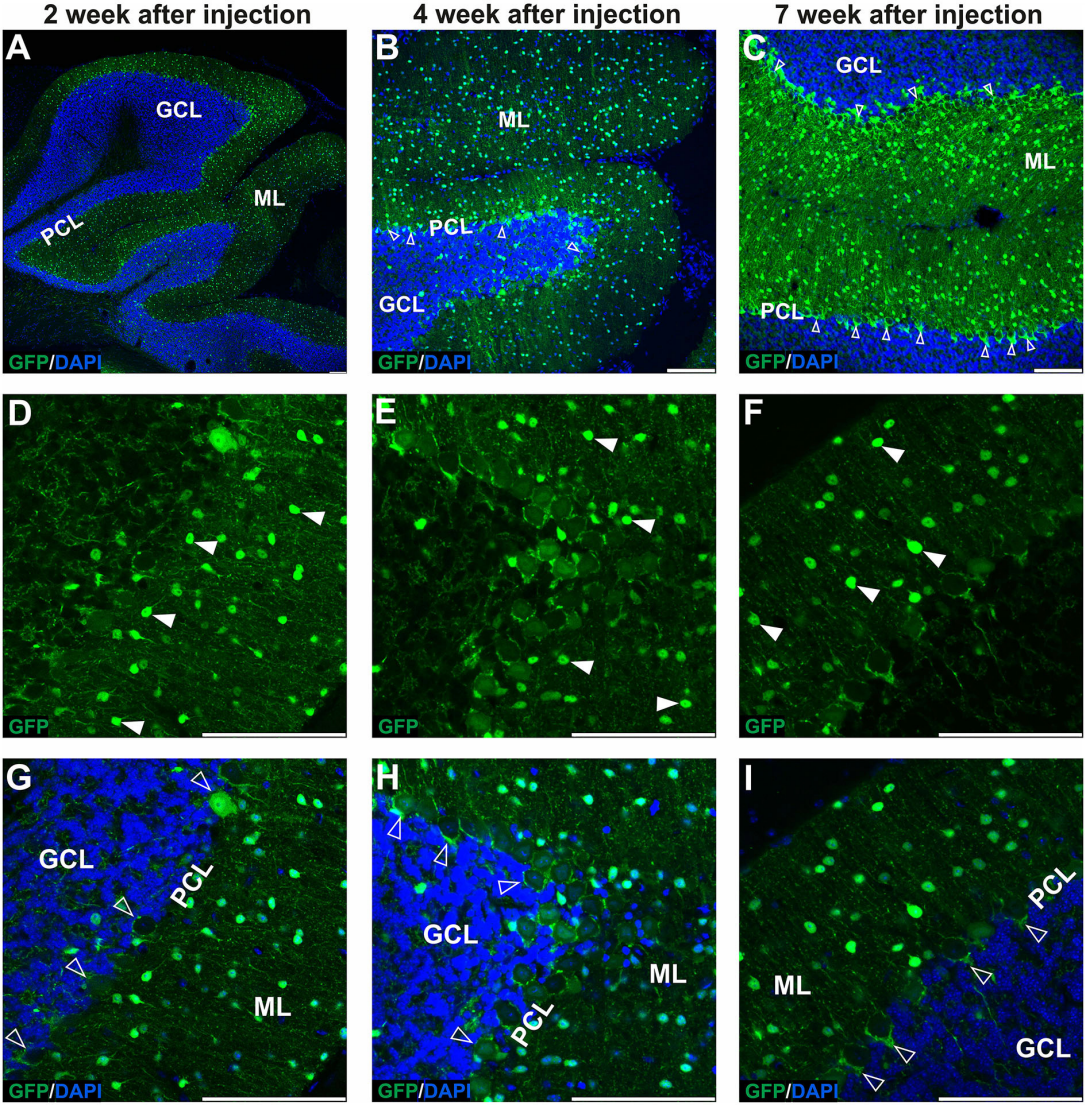
The early transduction phase of the mouse cerebellum after injection of the eGFP-encoding AAVrh10 vector into cerebellar lobules. The transduction pattern was detected by imaging eGFP+ cells at 2 **(A,D,G)**, 4 **(B,E,H)**, and 7 weeks post-injection **(C,F,I)**. The eGFP+ cells were observed in the molecular layer (arrowhead, **D–F**), which demonstrated small cell bodies and characteristic locations reminiscent of stellate cells. Importantly, no eGFP expression was observed in Purkinje cells. In addition, a strong eGFP+ signal was observed in characteristic structures called pinceau and wrapped around eGFP-negative Purkinje cell bodies (unfilled arrowhead, **B,C,G–I**). Such characteristic structures are long known exclusively as parts of the processes of basket cells. In summary, the transduction phase demonstrated transduction of the molecular cell layer and lack of transduction in Purkinje cell and granule cell layers. **(A–C,G–I)** eGFP green staining was merged with DAPI blue nuclear staining. Arrowheads: eGFP+ cells in ML. Unfilled arrowheads: pericellular baskets and pinceau; GCL, granule cell layer; ML, molecular layer; PCL, Purkinje cell layer. Coronal sections. Bars: 100 μm in the lower right corner of images. Purkinje cell and granule cell transduction: <1%; *N* = 3 animals, see Figure 8.

The GFP encoding AAVrh10 was double injected in the vicinity of ML of the cerebellum's lobules IV-V and lobule VIII (see Section Methods for stereotaxic coordinates). After 2, 4, and 7 weeks, the brains were cryosectioned (coronal), immunostained, and the fluorescent signal was detected using the Opera Phenix system. The transduction pattern at the early stage up to 7 weeks was characterized by the majority of the green fluorescent signal detected in ML of several lobules, and hardly any signal was present in GCL and PCL (<1% transduction; *N* = 3 animals; Figures 1, 8). The AAVrh10_eGFP molecular layer transduction pattern, which we could identify up to 7 weeks, was characteristic of specific eGFP-positive cell populations. According to the well-established cerebellar cellular composition and architecture, the eGFP+ cells in ML could be attributed to stellate and basket neurons. Basket and stellate neurons were represented as characteristic eGFP+ cells scattered along with ML (Figures 1D–I; arrowhead). The other eGFP+ signal was, at first glance, attributed to the bodies of Purkinje cells. However, closer examination of Z-stacks and high magnification revealed that the signal is distributed tightly around the body of Purkinje, showing pericellular baskets and forming specialized structures of basket cells, known as pinceau (Figures 1B,C,G–I; unfilled arrowheads) (Brown et al., 2019). Remarkably, the early stage of the AAVrh10_eGFP transduction showed hardly any transduction of the Purkinje cells (Figures 1G–I; unfilled arrowheads). We concluded that lobular injections of AAVrh10_eGFP up to 7 weeks in the mouse brain could be used as a paradigm for the transfection of ML cells but not Purkinje cells in the cerebellum.

**Figure 2:**
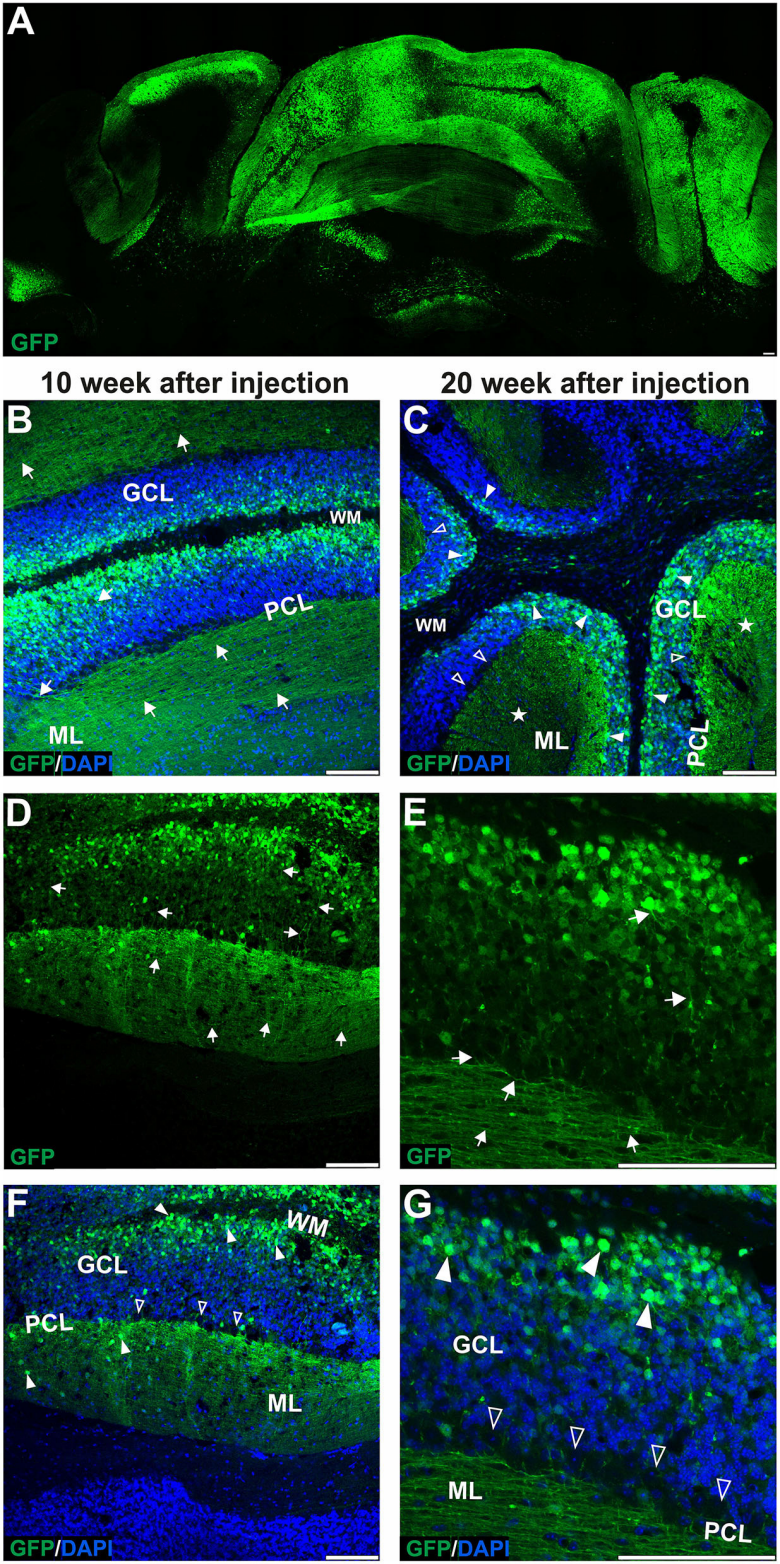
The late transduction phase of the mouse cerebellum after injection of the eGFP-encoding AAVrh10 vector into cerebellar lobules. The imaging of eGFP+ cells was performed after 10 **(B,D,F)** and 20 weeks after injection **(A,C,E,G)** and demonstrated densely packed round transduced cell bodies in the granule cell layer (arrowheads, **B,C,F,G**). Both 10 and 20 weeks after injection, there were characteristic eGFP-positive axons of granule cells running in the granule cell layer (arrows, D-E) and further eGFP-positive parallel fibers in ML (arrow, **B,D,E**, asterisks **C**). Similar to early transfection, the eGFP signal was not observed in the Purkinje cells (unfilled arrowheads indicate unstained PC bodies, **C,F,G**); however, few cells in the ML were still showing some eGFP signal. Two panels **(E,G)** show bodies and axons of granule cells under higher magnification. In summary, the late transduction phase using the AAVrh10 serotype demonstrated that eGFP+ signal shift results in almost exclusive transduction of granule cells and labeling of their proximal axons and distant parallel fibers. Unfilled arrowheads: Purkinje cell bodies. Arrows: eGFP expression in parallel fibers or axons of granule cells. Asterisks: parallel fibers on cross-section. GCL, granule cell layer; ML, molecular layer; PCL, Purkinje cell layer; WM, white matter. **(A,B,D–G)**: Coronal sections, **(C)**: Sagittal sections. Bars: 100 μm in the lower right corner of images. Granule cell transduction efficiency after lobular injections of AAVrh10 (late stage): 76.3% (*N* = 3 animals; see Figure 8 for histogram).

**Figure 3:**
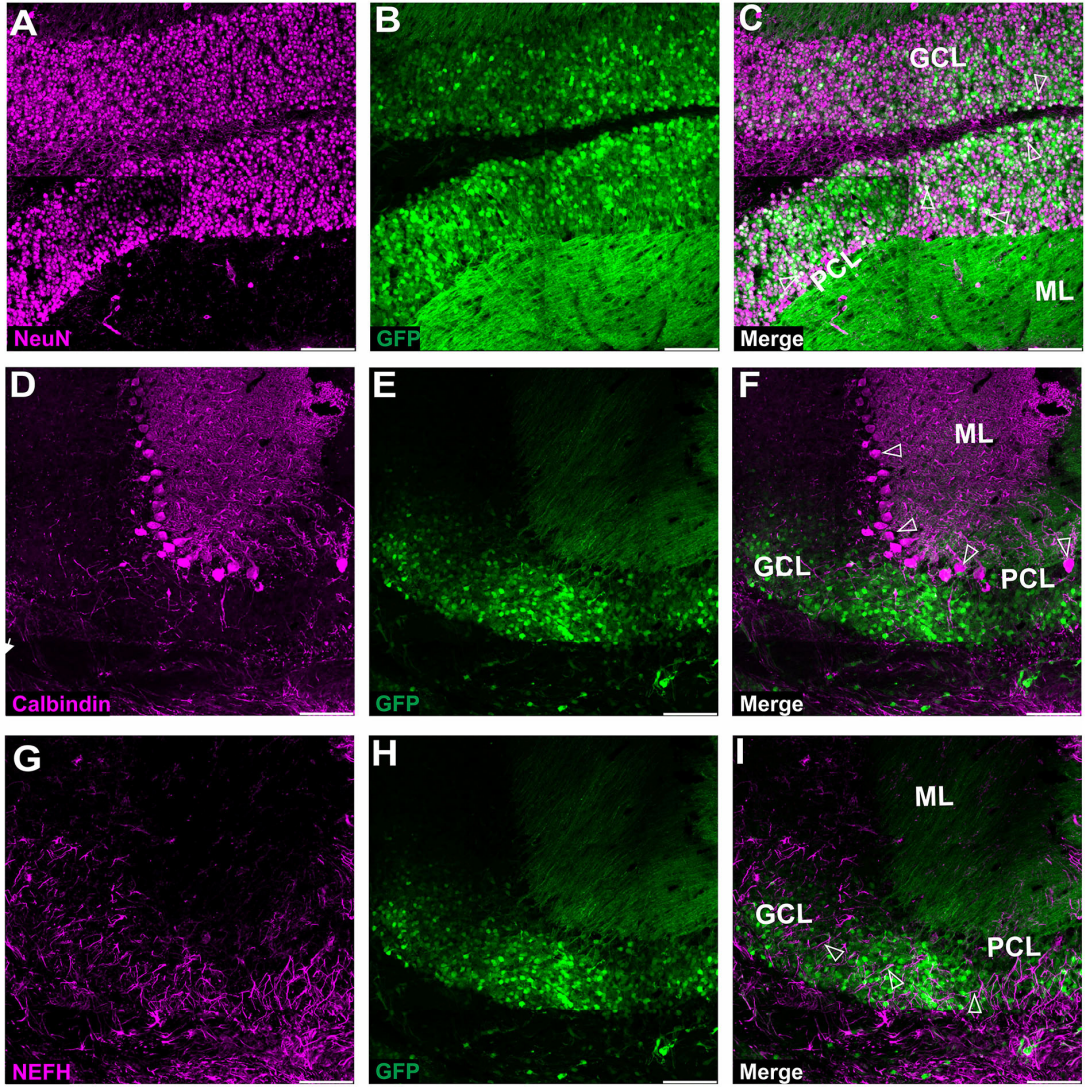
Fluorescent coimmunostaining of mouse cerebellar cryosections between 10 and 20 weeks after lobular AAVrh10 injections. eGFP green signal **(B,E,H)** was imaged together with NeuN (magenta) or calbindin 1 (magenta) or NEFH neurofilament (magenta). The granule cell marker, NeuN **(A)**, co-localized with green cells **(B)**, showing an overlayed white signal **(C)**. Immunostaining for Purkinje cell marker calbindin 1 **(D)** did not reveal any co-localization with eGFP signal **(F)**. The NEFH marker, which is known for staining Purkinje and basket cell processes in the cerebellum (Wiatr et al., 2021) did not co-localize with eGFP **(G)**. Unfilled arrowheads: co-localized in granule cells **(C)**; or Purkinje cell bodies **(F)**; or neurofilaments **(I)**. GCL, granule cell layer; ML, molecular layer; PCL, Purkinje cell layer. Coronal section. Bars: 100 μm in the lower right corner of images. A slight misalignment of the composite images in **(A–C)** panels is due to the optical properties of Opera Phenix. Images acquired from *N* = 3 animals.

**Figure 4:**
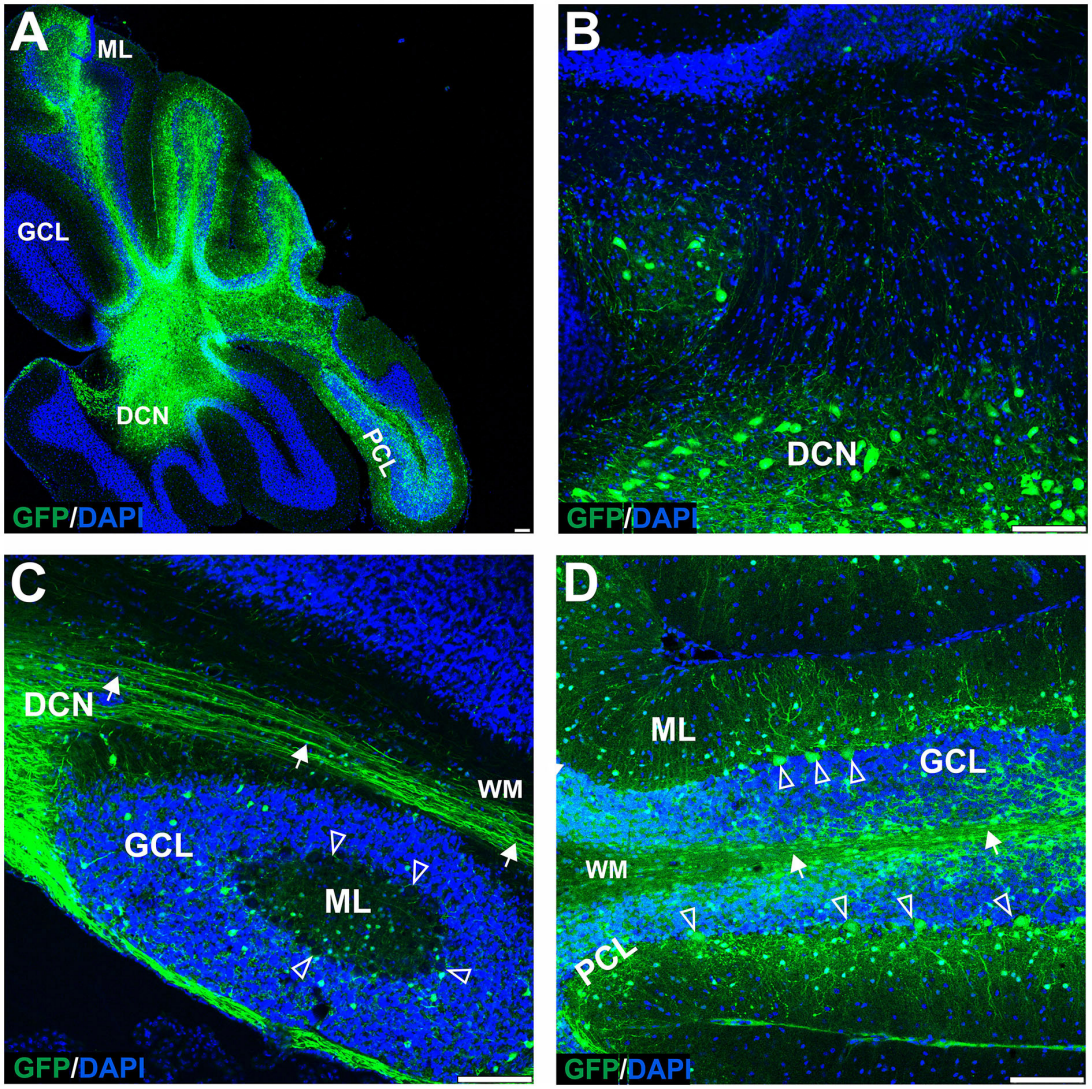
The transduction of the mouse cerebellum after injection of the eGFP-encoding AAVrh10 vector into deep cerebellar nuclei. Green eGFP+ signal was observed in deep cerebellar nuclei **(A,B)** and white matter **(A–D)**, in DCN neurons **(B)**, Purkinje cells, and cells of the molecular and granule cell layers **(D)**. Despite the selection of relatively prolonged time of observation (10 weeks post-injection) and the occurrence of intensive staining of the white matter fibers (likely Purkinje cell axons), the eGFP+ Purkinje cell bodies and dendrites did not occur very frequently. Unfilled arrowheads: Purkinje cell bodies. Arrows: eGFP expression in PC axons. DCN, deep cerebellar nuclei; GCL, granule cell layer; ML, molecular layer; PCL, Purkinje cell layer; WM, white matter. Sagittal sections. Bars: 100 μm in the lower right corner of images. Purkinje cell transduction efficiency at DCN injection with AAVrh10: 13% (*N* = 3 animals, see Figure 8).

**Figure 5:**
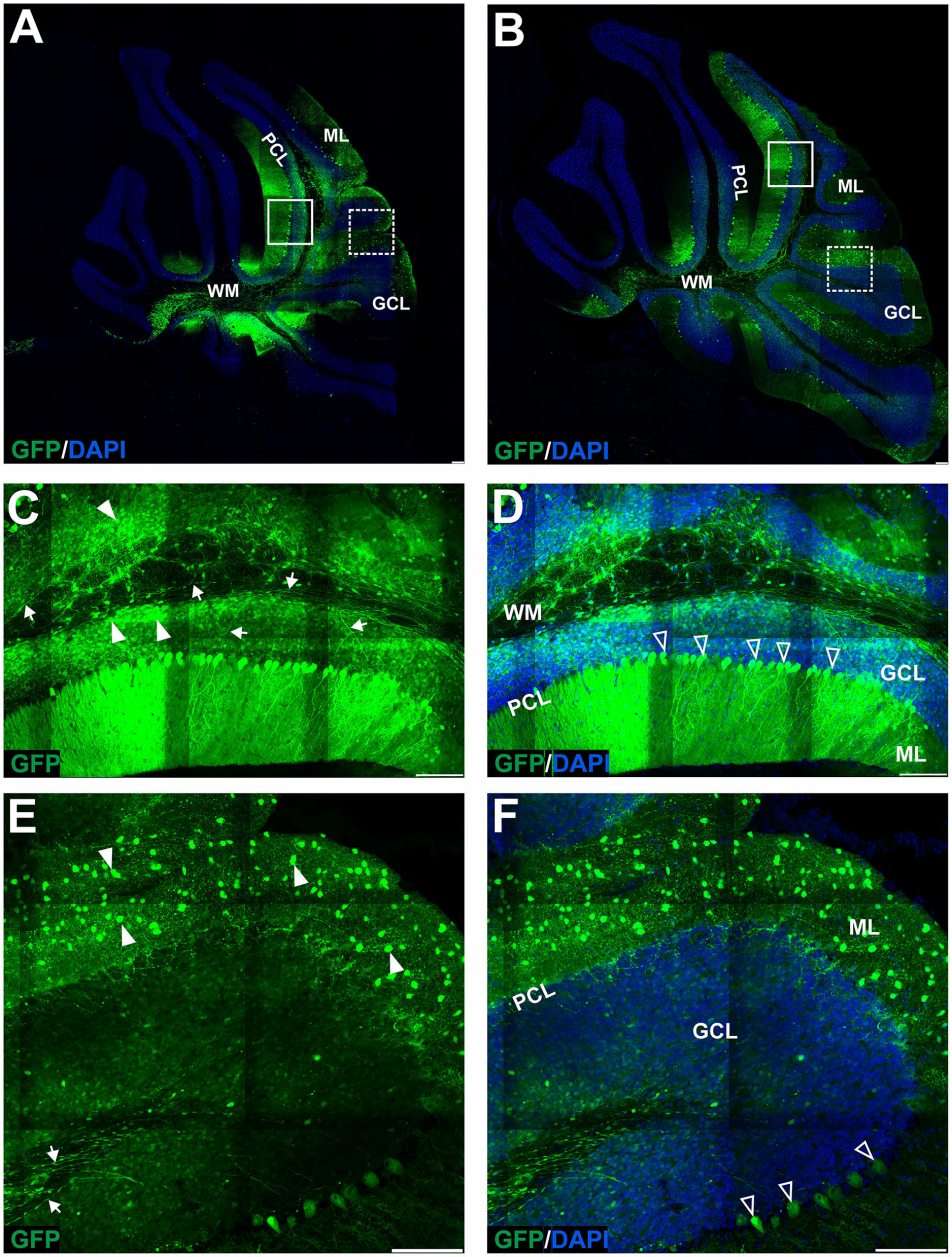
The transduction of the mouse cerebellum after injection of the eGFP-encoding AAV-PHP.eB vector into the cerebellar lobules. Green signal in eGFP+ cells on the sagittal sections was evident at 4 **(A,C–F)** and 7 **(B)** weeks after injection. Two transduction patterns were observed: pattern 1 indicated by the square **(A,B)**, and pattern 2 by dashed line square **(A,B)** and presented, respectively, in **(C–F)** (4 weeks). Both patterns demonstrated various types and counts of transduced cells in ML, PCL, and GCL. Unfilled arrowheads: eGFP-positive Purkinje cells. Arrowheads: eGFP-positive cells; Arrows: eGFP+ axons of PCs. GCL, granule cell layer; ML, molecular layer; PCL, Purkinje cell layer; WM, white matter. Sagittal sections. Bars: 100μ m in the lower right corner of images. Images acquired from *N* = 3 animals.

**Figure 6:**
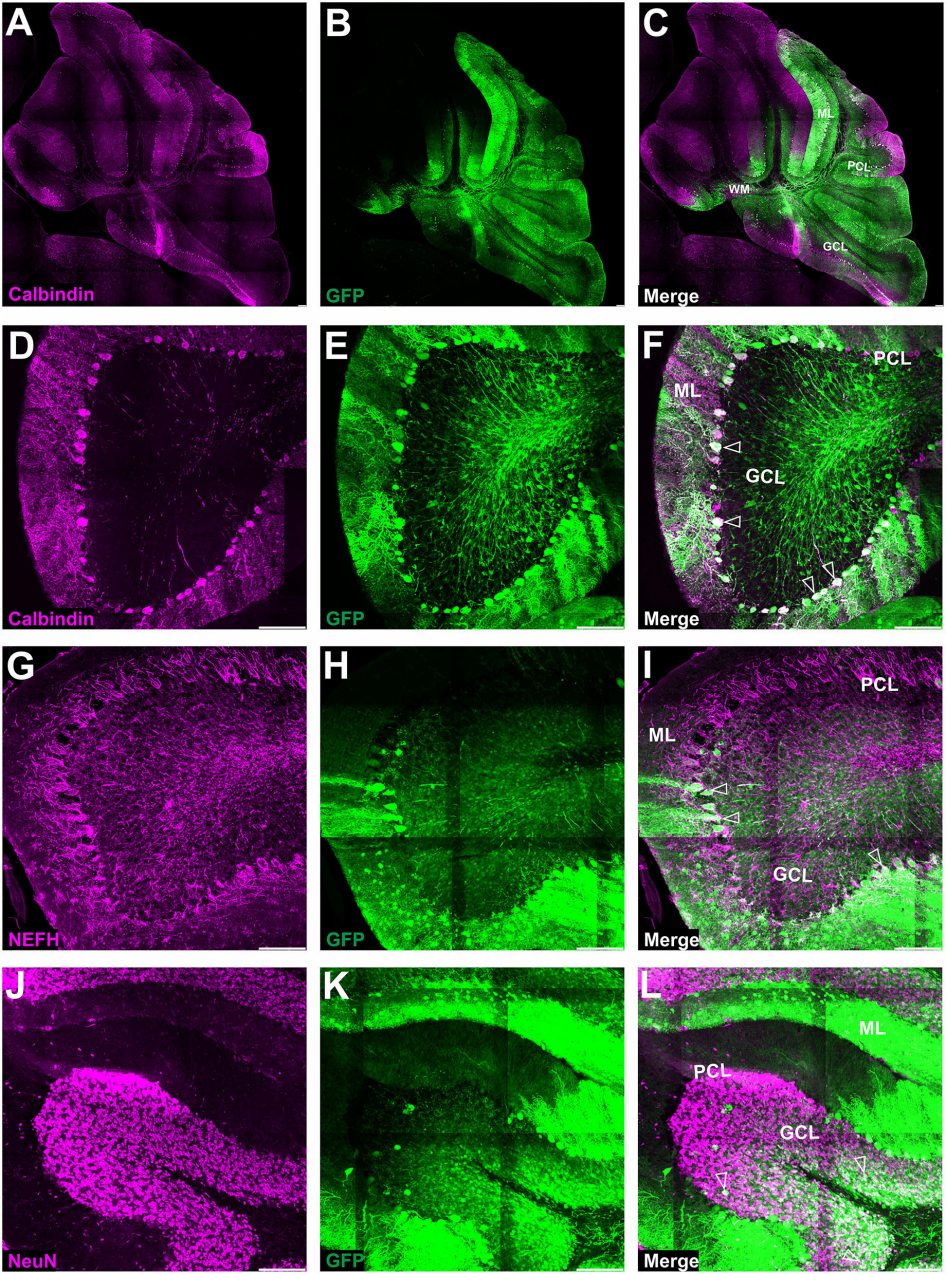
Fluorescent coimmunostaining of mouse cerebellar cryosections after lobular injection of eGFP-encoding AAV-PHP.eB. Green eGFP signal **(B,E,H,K)** was imaged together with calbindin 1 [magenta, **(C,F)**] or NEFH [magenta, **(I)**], or NeuN [magenta, **(L)**]. The co-localization signal (white) is present for all types of cells in the cerebellum, indicating the transduction of Purkinje cells [calbindin 1; **(A,D)**], basket cells [NEFH visible in Purkinje pericellular baskets; **(G)**] and granule cells [NeuN in GCL; **(J)**]. Unfilled arrowheads: Purkinje cell bodies **(C)** or pericellular baskets **(F)** or granule cells **(I)**. GCL, granule cell layer; ML, molecular layer; PCL, Purkinje cell layer. Micrographs containing only the eGFP channel were separately demonstrated for distribution evaluation. Sagittal section. Bars: 100 μm in the lower right corner of images. Images acquired from *N* = 3 animals.

**Figure 7:**
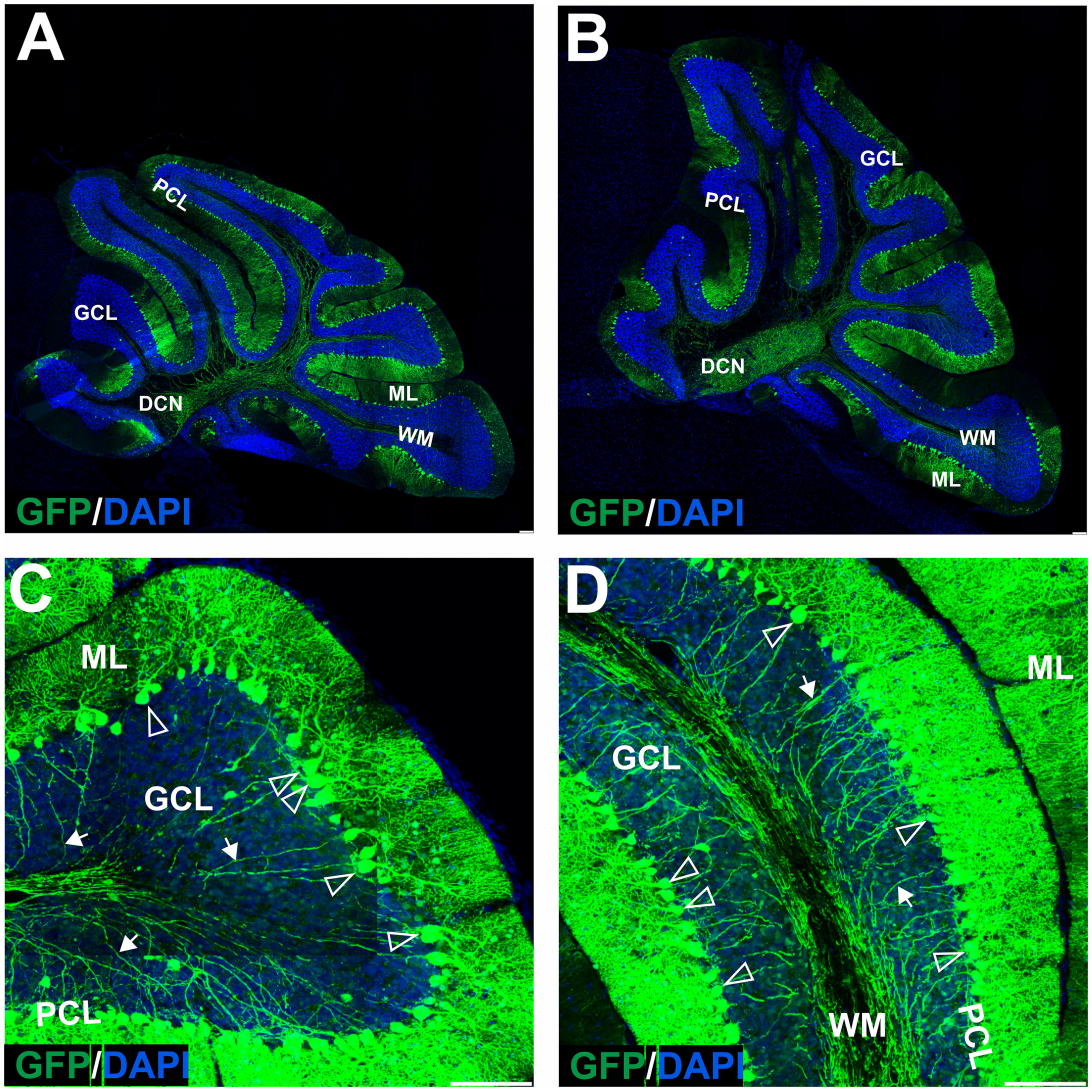
The transduction of the mouse cerebellum after injection of the eGFP-encoding AAV-PHP.eB into the deep cerebellar nuclei. A robust green signal has been observed throughout the cerebellar ML and was localized almost exclusively in Purkinje cells on the sagittal sections at 4 **(A,C,D)** and 7 **(B)** weeks after injection. Higher magnification of the transduction pattern was shown in **(C,D)**, 4 weeks after injection. eGFP signal was observed in all compartments of the PC, including bodies and dendritic trees (unfilled arrowheads), and axons (arrows). DCN, deep cerebellar nuclei; GCL, granule cell layer; ML, molecular layer; PCL, Purkinje cell layer; WM, white matter. Sagittal section. Bars: 100 μm in the lower right corner of images. Purkinje cell transduction efficiency at DCN injection: 93% (*N* = 3 animals, see Figure 8).

**Figure 8:**
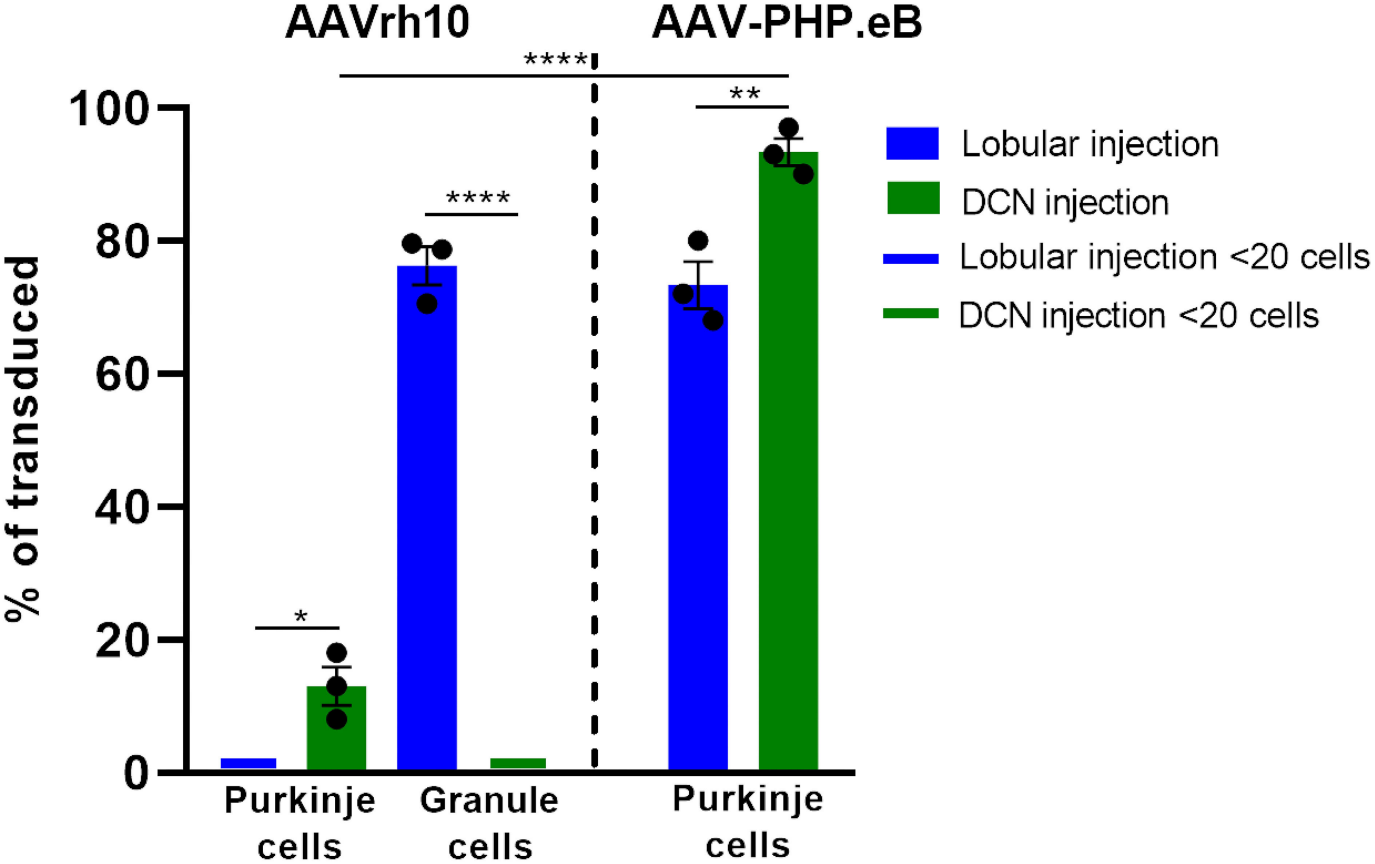
Histogram summarizing the transduction efficiency of Purkinje cells and granule cells after lobular and DCN injection of AAVrh10-eGFP and AAV-PHP.eB-eGFP. Purkinje cells transduction efficiency after AAVrh10 lobular vs. DCN injections was close to 1% vs. 13%, respectively (*N* = 3; difference between means 12% 1 2.887 SEM; *p* < 0.0142, *). Granule cell transduction efficiency after AAVrh10 lobular vs. DCN injections was 76% (late stage) vs. close to 1%, respectively (*N* = 3; difference between means 75% ± 2.895 SEM; t-test *p* < 0.0001, ****). Purkinje cells transduction efficiency after AAV-PHP.eB lobular vs. DCN injections was 73% (Pattern 1; note Figure 5) vs. 93%, respectively, AAV-PHP.eB (*N* = 3; difference between means 20% ± 4.069 SEM; t-test *p* < 0.008, **). Pattern 2 demonstrated hardly any transduction of Purkinje cells (see Figure 5). In addition, Purkinje cells transduction efficiency after injections in DCN with AAVrh10 vs. AAV-PHP.eB vector showed 13% vs. 93% (*N* = 3; difference between means 80% ± 3.528 SEM; t-test *p* < 0.0001, ****).

### The later stage of cerebellar transduction with AAVrh10_eGFP into lobules is characterized by GFP+ signal in axons and soma of granule neurons

Upon further investigation, we noticed that the cellular pattern of eGFP expression in the cerebellum changes 10 and 20 weeks post-AAVrh10_eGFP injection into lobules. Between 10 and 20 weeks, the eGFP signal was also evident in the granule cell layer, which demonstrated intense green staining of their bodies (Figure 2). Moreover, at 10 and 20 weeks post-lobular injections, the eGFP green signal was evident in axons coming out of eGFP+ granule cell bodies, passing through the Purkinje cell layer, reaching the molecular layer, and running horizontally along with the ML (Figures 2B,D,E). At the late stage after lobular injections, we found that the transduction of granule cells spanned across almost the entire granule layer on multiple cerebellar sections, indicating wide AAV distribution. The transduction efficiency in granule cell layers was high and reached 76.3% (lobular injection, *N* = 3 animals; Figure 2) vs. close to 1% (DCN injection, *N* = 3 animals; Figure 4, note Figure 8). Due to very high granule cell density and high eGFP+ signal, the estimation was done by measuring the fluorescent area and confirmed by manual cell counting. Interestingly, the eGFP signal in ML cell bodies started to disappear with increasing time post-injection. However, a small number of eGFP+ cell bodies in ML was still noticeable after 10 and 20 weeks. Moreover, eGFP signal also disappeared from the pinceau (Figures 2C,F). The eGFP green axons of granule cells running as parallel fibers along ML were visualized by performing the coronal sections (Figures 2B,D–G). On sagittal sections, the eGFP+ parallel fibers appear as dots (indicated by an asterisk in Figure 2C). Noteworthy, the eGFP expression after 10 and 20 weeks was more intense in granule cells in the more inner GCL, adjacent to the white matter (known as phylogenetically oldest granule cells) (Figures 2F,G, arrowheads). We have confirmed the identity of the AAVrh10_eGFP-transduced cells by immunochemistry. The eGFP-positive granule cells were robustly stained with GC marker NeuN showing co-localization (Figures 3A–C; co-localization: white). The markers of other cell types did not co-localize with the GFP+ staining (Figure 3). In particular, calbindin 1 staining revealed bodies, dendritic trees, and axons of Purkinje cells (Figures 3D–F), which showed no eGFP signal in these structures after lobular AAVrh10_eGFP. The neurofilament NEFH+ processes in ML and GCL were eGFP-negative, indicating the lack of transduction of basket cells and PC processes (Wiatr et al., 2021) at the late stage (Figures 3G–I).

### AAVrh10_eGFP delivery into DCN results in eGFP+ axonal tracts but a lower number of eGFP+ Purkinje cell bodies and dendrites

The lack of transduction of Purkinje cells by the lobular injection prompted us to look for a new mode of AAVrh10_eGFP vector delivery. The lobular injections of AAVrh10_eGFP initially resulted in ML transduction, and later we observed displacement of eGFP signal from ML to granule cell axons and transduction of the deeper cerebellar layers. The transduction pattern that changes in time and indirect transduction of cell bodies distant from the injection site indicates the retrograde transport of vectors along axons. Since the axons of PC follow white matter tracts and form synapses on neurons in DCN, we hypothesized that efficient local transduction of PC could be achieved by AAVrh10_eGFP delivery by injection into the DCN and the transport of the vector along the axons. We performed the injections and demonstrated that the DCN was successfully targeted with AAVrh10_eGFP, showing intense eGFP+ signal 10 weeks post-injection (Figure 4A). Moreover, the eGFP signal was detected far from the injection site (Figures 4A,B) along the axons (Figure 4C) and also in several Purkinje cell bodies (Figure 4D). DCN neurons (Figure 4B) and axons in WM (Figures 4C,D, arrow) were transduced. Purkinje cells transduction efficiency after AAVrh10 lobular vs. DCN injections was close to 1% vs. 13%, respectively (*N* = 3; difference between means 12% ± 2.887 SEM; *p* < 0.0142).

### AAV-PHP.eB_eGFP produces efficient transduction of cerebellar Purkinje cells after systemic retro-orbital delivery

The AAV-PHP.eB serotype can cross the blood–brain barrier and demonstrate efficient transduction of cerebellar Purkinje cells after systemic retro-orbital injection (Hordeaux et al., 2019). Also, in our experiments, 2 weeks after systemic injection of AAV-PHP.eB_eGFP, a very bright eGFP signal was revealed in Purkinje cells. The eGFP was present in all PC cellular compartments (Supplementary Figures S1A–C), including the soma and dendritic trees (Supplementary Figure S1C; unfilled arrowhead), and axons (Supplementary Figure S1B; arrow), passing through the white matter and granule layer. The transduction of PC with the AAV-PHP.eB_eGFP was relatively selective to PC, indicating a high tropism of the serotype toward PC. Interestingly, when administered retro-orbitally, no granule cells or their axons were targeted by the AAV-PHP.eB_eGFP serotype.

### Lobular injections of AAV-PHP.eB_eGFP produce an inconsistent pattern of EGFP+ cells in ML and eGFP+ Purkinje cells

Since systemic AAV-PHP.eB administration demonstrated homogeneous PC transduction with a strong eGFP+ signal, we investigated the possibility of local delivery of the serotype to avoid the transduction of other brain regions and induce the transduction of the Purkinje cells. Therefore, we have double injected the AAV-PHP.eB_eGFP in the vicinity of ML of the cerebellar lobules of the vermis (Section Methods) and collected the cerebellum after 4 (Figure 5A) and 7 (Figure 5B) weeks and observed two distinct patterns of lobular transduction. The first pattern consisted of lobules with the eGFP expression in Purkinje cells (cell soma, dendrites, and axons) and granule cells (Figures 5A,B; square). The second transduction pattern present in some lobules was visible as transduction of the molecular layer cells with the low number of transduced PCs (Figures 5A,B; square with dashed line). Representative examples of both transduction patterns at week 4 are shown in Figures 5C,D (pattern 1) and 5E-F (pattern 2). We performed co-localization immunostaining (white signal) which identified the eGFP-positive Purkinje cells (calbindin 1), neurofilament (NEFH), and granule cells (NeuN) after lobular AAV-PHP.eB_eGFP delivery (Figure 6). The NEFH immunostaining (neuronal processes) showed numerous neurofilaments located in GCL and ML and close to the PC layer showing some co-localization with eGFP (Figure 6I). Overall, the local lobular delivery of the AAV-PHP.eB_eGFP demonstrated an irregular pattern of transduction, where the eGFP signal was located in many cell types but with varying compositions of transduced cells. Such transduction pattern also demonstrates that the CMV promoter used for eGFP expression in AAV-PHP.eB construct is very effective in all cell types of the cerebellum. The transduction efficiency of Purkinje cells by lobular injections was very low using AAVrh10, and only single Purkinje cells were transduced (~1%) on all slides. In the case of the AAV-PHP.eB lobular injections, the transduction efficiency was inconsistent, showing 73% transduction efficiency of Purkinje cells (white square: pattern 1), and in another location, only the single PCs were transduced (dashed line white square: pattern 2) (Figures 5, 8). Therefore, the values of transduction efficiency of PCs using lobular injection for both AAVrh10 and AAV-PHP.eB is very difficult to determine consistently.

### Local DCN delivery of AAV-PHP.eB_GFP reveals uniform transduction of Purkinje cells

In the earlier experimental set with AAVrh10_eGFP and DCN injections, we demonstrated the feasibility of delivering vectors *via* Purkinje cell axons. To reproduce the transduction paradigm with the AAV-PHP.eB serotype, we performed local injections to deliver the vector to the PC terminals in DCN. The AAV-PHP.eB_eGFP was delivered into the left and right DCN by direct injection. As for AAVrh10, AAV-PHP.eB_eGFP efficiently transduced DCN neurons at 4 and 7 weeks post-injection (Figures 7A,B). The cerebellar lobules demonstrated intense transduction both at 4 and 7 weeks post-injection (Figures 7A,B). eGFP signal (Figures 7C,D) was observed almost exclusively in the soma of Purkinje cells and their dendritic trees (unfilled arrowheads) and axons running from PC bodies through the granule cell layer and WM. The transduction pattern of Purkinje cells after injection of AAV-PHP.eB_eGFP in DCN did not differ in intensity between 4 and 7 weeks (Figures 7A,B). The transduction efficiency of Purkinje cells after AAV-PHP.eB_eGFP in DCN vs. lobular injection was 93 vs. 73%, respectively (*N* = 3 animals; difference between mean values, 20 ± 4.069% SEM; *t*-test, *p* < 0.008; Figure 8). In addition, the transduction efficiency of Purkinje cells after DCN injection was 13% for AAVrh10 vs. 93% for AAV-PHP.eB (*N* = 3 animals; difference between mean values, 80 ± 3.528% SEM; *t*-test, *p* < 0.0001, Figure 8). Remarkably, the transduction of Purkinje cells by AAV-PHP.eB into DCNs was very stable, showing the same eGFP transduction pattern as early as 2 weeks until at least 7 weeks (Supplementary Figure S3), also demonstrating silencing resistance of the CMV promoter in our AAV-PHP.eB_eGFP setup. We conclude that DCN injection of AAV-PHP.eB_eGFP produces transduction of Purkinje cells in the cerebellum and can be an efficient candidate paradigm to deliver local gene therapy into these cells.

### CMV and PGK promoter constructs induce a comparable and high level of eGFP fluorescent signal both in Purkinje and granule cells

The potency of the CMV and PGK promoters in inducing the expression of eGFP protein in the transfected Purkinje and granule cells was assessed by analyzing the fluorescent eGFP signal in single cells. The Purkinje and granule cell bodies with clear eGFP labeling (at least image intensity value of 80 above BG) and well-spaced from each other were selected. The images of cell bodies were collected using the same intensity settings on Opera Phenix. Subsequently, the ROI was placed around the cell body, and the pictures were measured to acquire integrated optical density (Fiji/ImageJ). Supplementary Figure S2 demonstrates Purkinje cells (A) and granule cells (B) transfected with AAVrh10 (PGK promoter), while (C,D) demonstrate the integrated optical density values for Purkinje and granule cells, respectively. The results indicate that both cassettes containing PGK and CMV promoters induce high levels of eGFP expression both in PCs and GCs.

## Discussion

The AAV vectors are increasingly used as effective therapeutic tools for delivering gene therapies into the central and peripheral nervous system (Connor et al., 2016; Tervo et al., 2016). However, future precise modalities will require cell-specific targeting by AAVs to minimize the risk of side effects. Currently, the AAV specificity for brain cell types largely relies on controlling the expression using dedicated neuronal or glial promoters (Griffin et al., 2019; Hoshino et al., 2021). However, the promoter strategy may not be sufficient to avoid off-targeting other cells and prevent inflammatory response, genome integration, or other effects related to systemic or whole-brain delivery (Timmers et al., 2020). The current delivery issues include the undesired targeting in peripheral tissues, such as the liver and blood, in case of systemic injection of BBB-permeable vectors (Albright et al., 2018; Liu et al., 2021; Sherafat). Therefore, the cell specificity of the AAV serotypes remains to be precisely explored in the context of targeted delivery of the therapies only to the cell types involved in the pathogenic processes. In particular, the existing AAV technologies and serotypes allow for cell specificity by using the neural network's architecture and the delivery targeted at the localization of neuronal terminals. Among the available serotypes, the AAVrh10 and AAV-PHP.eB are very promising because of their previously documented features, such as transduction *via* retrograde transport, transduction of various cell types, including glia and neurons such as cerebellar Purkinje cells (Petrosyan et al., 2014; Tanguy et al., 2015), and BBB permeability in the case of PHP.eB (Mathiesen et al., 2020; Liu et al., 2021). Moreover, the affinity of AAV serotypes to glutamatergic or GABAergic neuronal main types and their subtypes must be determined.

The cerebellum is used as the choice structure in anatomical, functional, and developmental CNS studies. Its structure and cellular composition are well-defined to the point that almost all neural cell types can be identified relatively precisely, based mainly on eGFP to visualize the morphology and cellular localization. The cerebellum contains excitatory (glutamatergic) and inhibitory (GABAergic) neuronal types forming neuronal networks, which are well investigated (Gilkes et al., 2016; Tervo et al., 2016). Moreover, the cerebellum is a brain structure where the cell types are severely affected in many neurodegenerative diseases, including spinocerebellar ataxias, Huntington's disease, and other neurological disorders, such as autism spectrum (Wiatr et al., 2019; Tereshchenko et al., 2020; Cook et al., 2021; Niewiadomska-Cimicka et al., 2021). In particular, Purkinje cells are probably the most affected cell type by neurodegeneration in the cerebellum. Therefore, they may be important cellular targets to deliver gene therapies by AAV vectors.

We found that the injection of the AAVrh10 vector into the cerebellar lobules in the vicinity of the ML transduces stellate and basket cells; however, the Purkinje cells were not transduced in such an approach. Both transduced cell types can be easily recognized by their morphology, where the eGFP-positive basket cells form the characteristic tips called pinceau (Zhou et al., 2020) and wrap the eGFP-negative body of Purkinje cells. The transduction of stellate and basket cells started to disappear 7–10 weeks after AAVrh10 lobular injection. Around the same time, the granule neuron cell soma and axons began to show the eGFP+ signal, still without any sign of Purkinje cell transduction (Figures 1, 2). The observed transduction pattern of the ML and the observed “shift” to granule cells after lobular delivery cannot be attributed to the transport of eGFP protein itself to granule cells by an undefined mechanism. Detection of such transported protein would be significantly affected by the 26-h half-life of the eGFP in the cell, which was determined in the presence of translation inhibitors (Corish and Tyler-Smith, 1999; Danhier et al., 2015; Trauth et al., 2020). The half-life of 26 h demonstrates that the eGFP protein is relatively stable compared to other cellular proteins; however, such a half-life still excludes that the eGFP protein that undergoes transport will be stable and detected over prolonged periods of weeks after it decreases in ML +eGFP cells. Therefore, the disappearance of the eGFP signal in a cell is inevitably connected with the lack of further production of eGFP and the lack or suppression of genetic AAV material in such cells. A more plausible explanation is the retrograde capabilities of AAVrh10 and the transport of capsid, which were repeatedly reported (Wang and Zhang, 2021). Moreover, stellate, basket, and granule cells have axons located in ML, and hence are close and accessible to the lobular injection site of AAVrh10. In addition, the dendrites of the basket, stellate, and PCs form synapses with GC parallel fibers (Prestori et al., 2019). In contrast, axon terminals of Purkinje neurons are located in DCN, and hence are inaccessible for lobular injections of AAVrh10. Therefore, we placed the AAVrh10 at DCN (Figure 4) at PC axonal terminals, which produced robust transduction of several PCs. Such transduction of PC in DCN setup also demonstrates that PGK promoter driving eGFP is active in PC. Therefore, the lack of PC transduction by AAVrh10 in lobular injections is not due to the inactivity of PGK. In general, the PGK promoter is very effective in driving eGFP in PC by lentiviral cassette (Bosch et al., 2014).

In our experimental setup, we used the AAV-PHP.eB capsid combined with the eGFP payload driven by the CMV promoter. The use of CMV-driven promoters in selected applications is controversial, sometimes leading to difficulties in the interpretation of results. Previously, the reason was the instability and silencing of the resulting expression reported for the first generation of CMV promoters. The CMV promoter was discovered and patented over 30 years ago (Thomsen et al., 1984; Stinski, 1995). Later, several not very dated works, indeed, reported on the silencing of the CMV promoter (Schmidt et al., 1990; Paterna et al., 2000; Villuendas et al., 2001; Bauer et al., 2005). A crucial issue reported formerly was its silencing in the stem cells or during differentiation, hence indicating problematic use in the generation of transgenic mice (Meilinger et al., 2009; Herbst et al., 2012). Early versions of the CMV promoter were unsuitable for gene therapy because they were active only for 7–14 days (Löser et al., 1998). Since then, many recombinant, enhancer-containing (CMV and WPRE enhancers) hybrids and even silencing-resistant versions of CMV promoters were published and patented as early as 2011 (Yang and Mariati, 2015). Probably because of these efficient second-generation CMV tools, including resistance to silencing in stem cells, the promoter's use is now very prominent, including in transgenic mouse models. The enhancer-containing and hybrid CMV promoters also paved the way for AAV-based therapies, such as Glybera, Luxturna, and Zolgensma. In case of danger of quick silencing, the CMV elements would probably be avoided for use in gene therapies. We also observed stable eGFP expression from 2 to at least 7 weeks post-injection and beyond, indicating enhanced CMV promoter use in our AAV-PHPeB-containing CMV enhancer (Supplementary Figure S3). The level of expression and duration were not achievable with the previous generation of CMV promoters. Moreover, both the PGK and CMV promoters, which are present in our AAV cassettes, induce intensive and comparable eGFP expression in both Purkinje and granule neurons (Supplementary Figure S2).

**Figure 9:**
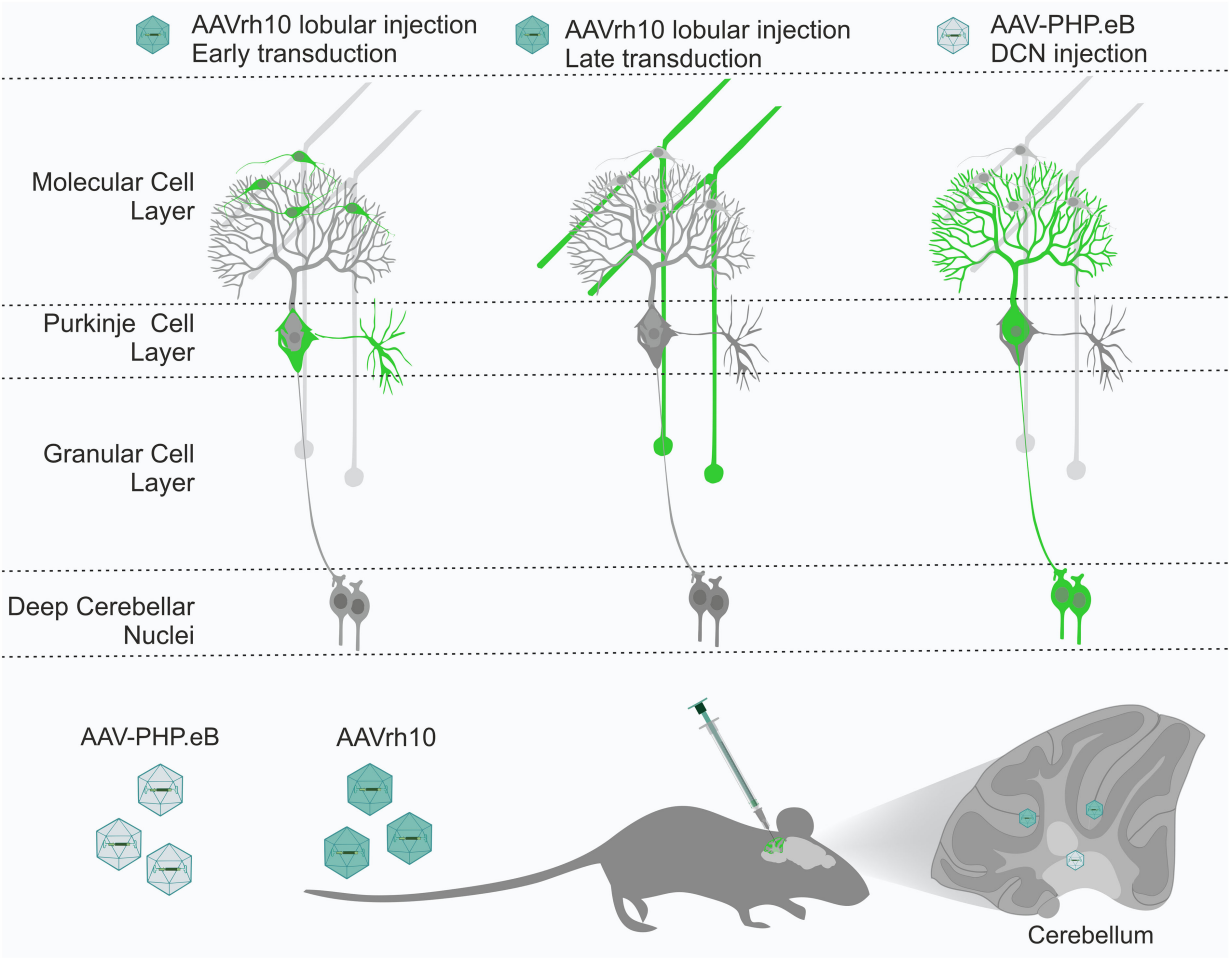
The cerebellum has several layers and structures, with many cell types, including Purkinje cells, granule neurons, stellate neurons, and basket neurons. All cell types are tightly interconnected by their axonal and dendritic processes, forming a perfect framework for retrograde delivery of AAV-based therapies to cell types of interest. We demonstrated that the AAVrh10 and AAV-PHP.eB viral particles placed in the vicinity of the axonal terminals by targeted brain injections into lobules, and deep cerebellar nuclei (DCN) can selectively transduce neuronal types of cerebellar networks, such as Purkinje cells and granule cells. AAVrh10 injected into lobules produces early transduction in stellate and basket cells. Subsequently, AAVrh10 retrogradely transduces granule cells. AAV-PHP.eB injected into DCN at the axonal terminals of Purkinje cells produces their selective transduction. Therefore, both serotypes can be used in a retrograde mode for selective transduction of major neuronal types in the cerebellum for effective and selective gene delivery. DCN, deep cerebellar nuclei.

In summary, the transduction of neurons with the AAVrh10 serotype is most successful when the vector can reach axonal terminals, which may activate its reported retrograde capabilities. Placing the AAVrh10 vector near the soma and dendritic tree is not sufficient for PC transduction. The indirect transduction of CNS neurons that use the AAV-based retrograde capabilities is highly promising as a therapeutic tool (Bravo-Hernandez et al., 2020; Wang and Zhang, 2021). In particular, the AAV9 was recently used for the transduction of motoneurons, which possess very long axons and are the target cell type in ALS (Bravo-Hernandez et al., 2020). The derivative of AAV9, AAV-PHP.eB, also produces transduction of Purkinje cells when delivered into the blood by retro-orbital injection, where we have seen almost complete transduction of these cells in the mouse cerebellum; however, no transduction of granule cells occurred. Therefore, we asked if we could reproduce such selective PC transduction by local delivery of PHP.eB. The lobular delivery produced inconsistent results with various types of cells transduced in different lobules of the cerebellum (Figure 5). However, the PHP.eB injected into the DCN at the axonal terminals of Purkinje cells produced uniform and selective transduction of Purkinje cells after 4 weeks (Figure 7). We conclude that the retrograde mode of transduction is very selective in the case of cerebellar cell populations. When placed in the vicinity of axonal terminals, the AAVrh10 and AAV-PHP.eB are complementary in targeting the most critical cell types in the cerebellum (Figure 9). Moreover, the receptors of both vectors need to be precisely investigated in the future. Both vectors may be used as a pair for the mutual control of the therapeutic effect and the side effects of candidate gene therapies in case of targeting unwanted cell types in the cerebellum. Our *in vivo* strategies are suitable for further pre-clinical and clinical protocol development for gene delivery to granule cells by AAVrh10 and Purkinje cells by AAV-PHP.eB.

## Materials and methods

### Generation of AAV particles and internal quality control assays

AAVrh10 particles were produced and purified by the vector core of the Gene Therapy Laboratory (Inserm, Nantes, France). The AAV-PHP.eB particles were produced in The Molecular Biology and virus service at the Institute of Genetics and Molecular and Cellular Biology (IGBMC, Strasbourg, France). The titers assayed at production sites by qPCR were 3.5 × 10^12^ vg/ml and 2.2 × 10^13^ vg/ml for AAVrh10 and AAV-PHP.eB, respectively. Since the titers were established in two independent production facilities, we implemented a qPCR using eGFP primers (Reverse: GCTTGTCGGCCATGATATAGA, Forward: GACGACGGCAACTACAAGA) as laboratory internal quality control prior to the injections. In brief, samples of AAVs were pipetted from each lot of AAV in three technical replicates and denatured for 10 min at 95°C in a buffer containing 0.01% SDS, and slowly cooled in a thermocycler block. qPCR for eGFP cDNA was performed using the dilution series (each dilution differed 10×). The qPCR was performed at 95°C for 10 min, 40 cycles of 95°C for 15 s, and at 60°C for 1 min. The control at our laboratory confirmed the quality of the AAV preparations and confirmed the same differences in amplification efficiency (10^12^ vs. 10^13^ titer range). In addition, we directly measured the content of ssDNA using Invitrogen Qubit 4 Fluorometer and an ssDNA Assay Kit (Thermo Fisher Scientific, Waltham, MA, United States), and confirmed the ssDNA content equivalent to vector copies assayed at production.

The rate of injections was 0.3 μl/min, with a total volume of 6 μl per cerebellar lobule or DCN. The dose for all intraparenchymal injections was 4 × 10^9^ vg/mouse and 1.5 × 10^10^ vg/g for systemic retro-orbital injections (Boussicault et al., 2016). AAVrh10 constructs contained the expression cassette with the eGFP reporter gene, driven by the PGK promoter. The AAV-PHP.eB vector contains the expression cassette with the eGFP reporter gene under the CMV promoter.

### Animals

The C57Bl/6J genetic background was used. Two-month-old mice with an average weight of 20–25 g were bred in the Center for Advanced Technologies AMU animal facility in Poznań or in the Mouse Clinical Institute (Illkirch, France). Animals were maintained in individually ventilated cages in SPF conditions under a 12-h light-dark cycle and water and food *ad libitum*. The number of mice in the experiments was planned according to the 3R recommendations. Experiments were approved and monitored by the Local Ethical Commission for Animal Experiments in Poznan (Poland) and in Illkirch (France) (Agreement Numbers: #13869-2018021216211243v5).

### Stereotactic and systemic injections

The AAVs were injected into cerebellar vermis lobules or the DCN at a dose of 4 × 10^9^ vg per mouse. C57BL mice (male or female) were anesthetized with isoflurane and secured in a stereotaxic frame (RWD Life Science Co., LTD, Shenzhen, Guangdong, China or Leica Angle Two with atlas, Leica) with the tooth bar individually adjusted for even horizontal skull position. For lobular injections, two holes were drilled through the skull (#1: 0.0, −6.4, −1.6; #2: 0.0, −7.4, −1.9; ML/AP/DV). The stereotaxic coordinates for injections into DCN in the left and right hemisphere were −1.2, −6.4, −3.0 and 1.2, −6.4, −3.0 (ML/AP/DVs), respectively. The injection coordinates were determined using Leica Angle Two—ATLAS stereotactic software (MYNEUROLAB.COM) based on a previous study (Paxinos, 2001) and compared with Allen Mouse Brain Atlas (Lein et al., 2007) (https://mouse.brain-map.org/static/atlas). In addition, the precise injection point was also verified by comparing the stained cerebellar slices to the Paxinos (integrated with stereotaxic equipment) and referencing Allen Mouse Brain Atlas. In the case of DCN, the selected stereotaxic coordinates were experimentally validated using green dye (Sigma Aldrich, Darmstadt, Germany), and the listed DV coordinate represents the depth of injection measured from the skull surface (DVs). In total, 6 μl of viral vectors (3 μl per lobule or DCN) were infused using a Hamilton 10-μl syringe with a 26-gauge needle and an automated infusion pump system (KD Scientific Inc., Massachusetts, USA) at the infusion rate of 0.3 μl/min. The needle was left in place for 5 min after each infusion before retraction. The dorsal-ventral coordinate is the absolute value (Burlot et al., 2015).

The C57BL mice were anesthetized and placed on a heating pad for retro-orbital delivery. The injection of viral vector (1.5 × 10^10^ vg/g, on average 100 ul volume) into the retro-orbital sinus next to the eye was performed using a disposable syringe with a 30-gauge needle. The syringe with the needle was left in place for 2 min after infusion before retraction.

### Tissue preparation and immunofluorescence

Adult mice were sacrificed at 2, 4, 7, 10, and 20 weeks post-injection by terminal isoflurane and transcardially perfused with 30 ml of ice-cold PBS followed by 30 ml of ice-cold 4% PFA in 0.1 M phosphate buffer (pH 7.4). The brains were removed and post-fixed overnight in ice-cold PFA, and cryopreserved with graded sucrose (10–20–30%) over 72 h. Brains were embedded in O.C.T. tissue freezing medium (Leica Microsystems, Poland) and frozen on dry ice. The brains were cut using a cryostat at −20°, and 20 μm sagittal and coronal sections were collected on SuperFrost Plus slides (Thermo Fisher Scientific, Waltham, MA, United States). Sections were stored at −80°C and air-dried for 10 min before immunostaining (Gage et al., 2012). The sections were processed immediately. The sections were blocked *via* incubation in 4% normal goat serum in PBS for 1 h. For immunofluorescence analysis, the following primary antibodies were used: anti-GFAP (1:1,000, BD Pharmingen), anti-calbindin 1 (1:2,500, Frontier), anti-NeuN (1:100, Millipore), and anti-NEFH (RT97, Hybridoma, 1:50). The sections were subsequently incubated with secondary antibodies conjugated with AlexaFluor647 and Cy3 (1:500; Jackson ImmunoResearch; Suffolk, United Kingdom). After washing in PBS, sections were counter-stained with Hoechst33342 (Sigma Aldrich, Darmstadt, Germany), mounted, and coverslipped with Fluoroshield (Fluoroshield histology mounting medium, Sigma Aldrich, Darmstadt, Germany). Confocal images were acquired using the microscope system Opera LX (PerkinElmer, Waltham, Massachusetts, USA) using ×20 air and ×40 water objectives, 100 ms exposure time, and 50–70% laser power. Images were processed using ImageJ software (National Institutes of Health, Bethesda, MD, USA). Statistical analysis was done using GraphPad Prism 5. All *p*-values were calculated by using a *t*-test for unpaired samples with Welch's correction. The graphs are presented as mean values with a standard error of the mean (SEM).

## Data availability statement

The original contributions presented in the study are included in the article/Supplementary material, further inquiries can be directed to the corresponding author.

## Ethics statement

Experiments were approved and monitored by the Local Ethical Commission for Animal Experiments in Poznan (Poland) (agreement number: 56/2018; 64/2018) and in Illkirch (France) (agreement number: #19490-2019022709337150_v4).

## Author contributions

MF was responsible for the research concept and obtaining funding, conceived and supervised all experiments, and analyzed the data. MS and EJ designed and performed all experiments, including cerebellar injections, immunohistochemistry, imaging, image processing, and analysis of the data. ŻK-P contributed to injections and immunochemistry. AN-C designed and performed initial retro-orbital injection experiments. YT supervised experiments and obtained funding. MF, MS, and EJ wrote the manuscript. All authors contributed to the article and approved the submitted version.

## Funding

This work was supported by the Grant from the European Research Projects on Rare Diseases (JTC 2017) Grant from the National Center for Research and Development [Grant Numbers: ERA-NET-E-RARE-3/III/TreatPolyQ/08/2018 (to MF and YT) and ERA-NET-E-RARE-3/III/SCA-CYP/09/2018 (to MF)], National Science Centre, Poland (Opus Grant, Number: 2021/41/B/NZ2/03881 to MF), and a Grant of National Ataxia Foundation #688790, Pioneer SCA3/MJD Translational Research Award (to MF and YT).

## Conflict of interest

The authors declare that the research was conducted in the absence of any commercial or financial relationships that could be construed as a potential conflict of interest.

## Publisher's note

All claims expressed in this article are solely those of the authors and do not necessarily represent those of their affiliated organizations, or those of the publisher, the editors and the reviewers. Any product that may be evaluated in this article, or claim that may be made by its manufacturer, is not guaranteed or endorsed by the publisher.

## Supplementary material

Supplementary Figure S1 Transduction pattern 2 weeks after retro-orbital injection of AAV-PHP.eB_eGFP **(A–C)**. eGFP signal was observed in axons of PCs in white matter **(B)**, PC bodies, and dendritic trees **(C)**. Almost no eGFP was present in GCL. Unfilled arrowheads: Purkinje cells; unfilled arrows: axons of PCs. DCN, deep cerebellar nuclei; GCL, granule cell layer; ML, molecular layer; PCL, Purkinje cell layer; WM, white matter. eGFP was shown for distribution evaluation. Sagittal sections. Bars: 100 μm in the lower right corner of images. Images acquired from *N* = 3 animals.

Supplementary Figure S2 Comparison of the activity of AAV cassettes containing eGFP under the control of CMV and PGK1 promoter in Purkinje and granule cells. Purkinje and granule cells were assayed by analyzing the fluorescent integrated optical density (IOD; Fiji/ImageJ) of the eGFP signal in single cells. Ten eGFP-positive cell bodies of Purkinje cells for each AAVrh10 (PGK promoter cassette) and AAV-PHP.eb (CMV promoter cassette) show a high level of eGFP by IOD measurement **(A,C)**, and 10 cell bodies of granule cells for each cassette also show a high level of eGFP by IOD measurement **(B,D)**. Both cassettes containing PGK and CMV promoters induce high levels of eGFP expression, and the measured IOD expression level is comparable in both PCs **(C)** and GCs **(D)**.

Supplementary Figure S3 Transduction pattern showing eGFP fluorescent signal after 2, 4, and 7 weeks post-DCN injection with AAV-PHP.eB. The figure demonstrates similar eGFP fluorescent intensity in Purkinje cells of two representative images for each time point: 2 **(A,D)**, 4 **(B,E)**, and 7 weeks **(C,F)** post-DCN injections. GCL, granule cell layer; ML, molecular layer; PCL, Purkinje cell layer; WM, white matter. Sagittal sections. Bars: 100 μm in the lower right corner of images.
