# Supplementary figures and images for: Selective transduction of cerebellar Purkinje and granule neurons using delivery of AAV-PHP.eB and AAVrh10 vectors at axonal terminal locations
Source: Front Mol Neurosci. 2022 Sep 13;15:947490. doi: 10.3389/fnmol.2022.947490 (PMC9513253; doi:10.3389/fnmol.2022.947490)

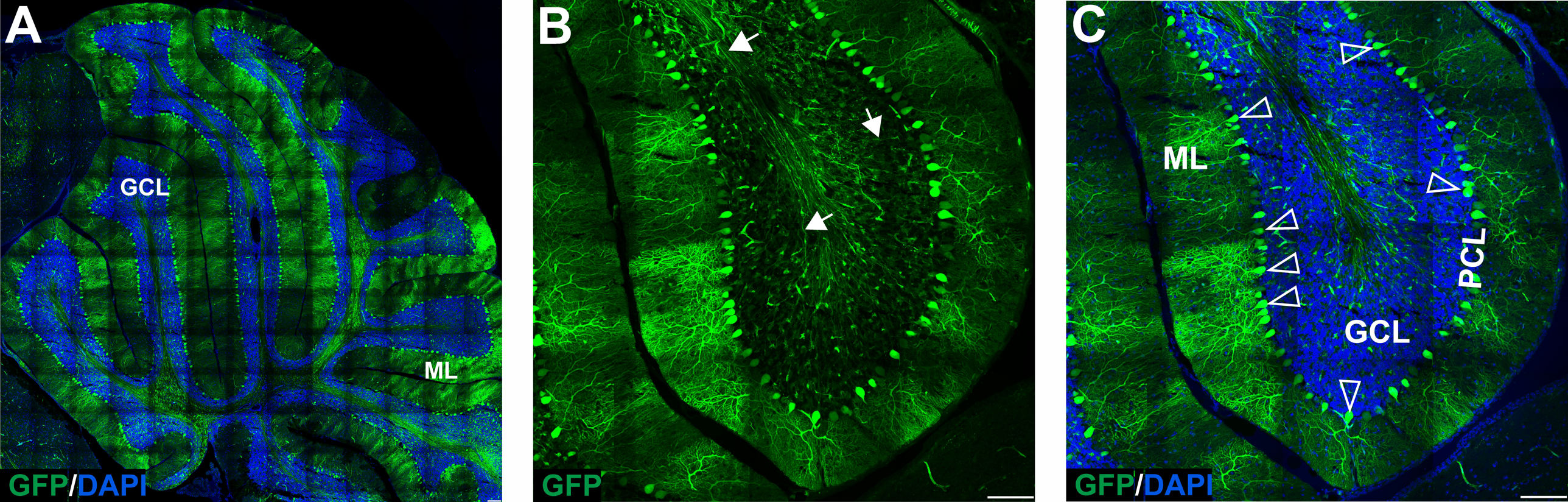

Supplement: Supplementary Figure S1 — Transduction pattern 2 weeks after retro-orbital injection of AAV-PHP.eB_eGFP (A–C). eGFP signal was observed in axons of PCs in white matter (B), PC bodies, and dendritic trees (C). Almost no eGFP was present in GCL. Unfilled arrowheads: Purkinje cells; unfilled arrows: axons of PCs. DCN, deep cerebellar nuclei; GCL, granule cell layer; ML, molecular layer; PCL, Purkinje cell layer; WM, white matter. eGFP was shown for distribution evaluation. Sagittal sections. Bars: 100 μm in the lower right corner of images. Images acquired from N = 3 animals. [file Image_1.JPEG]

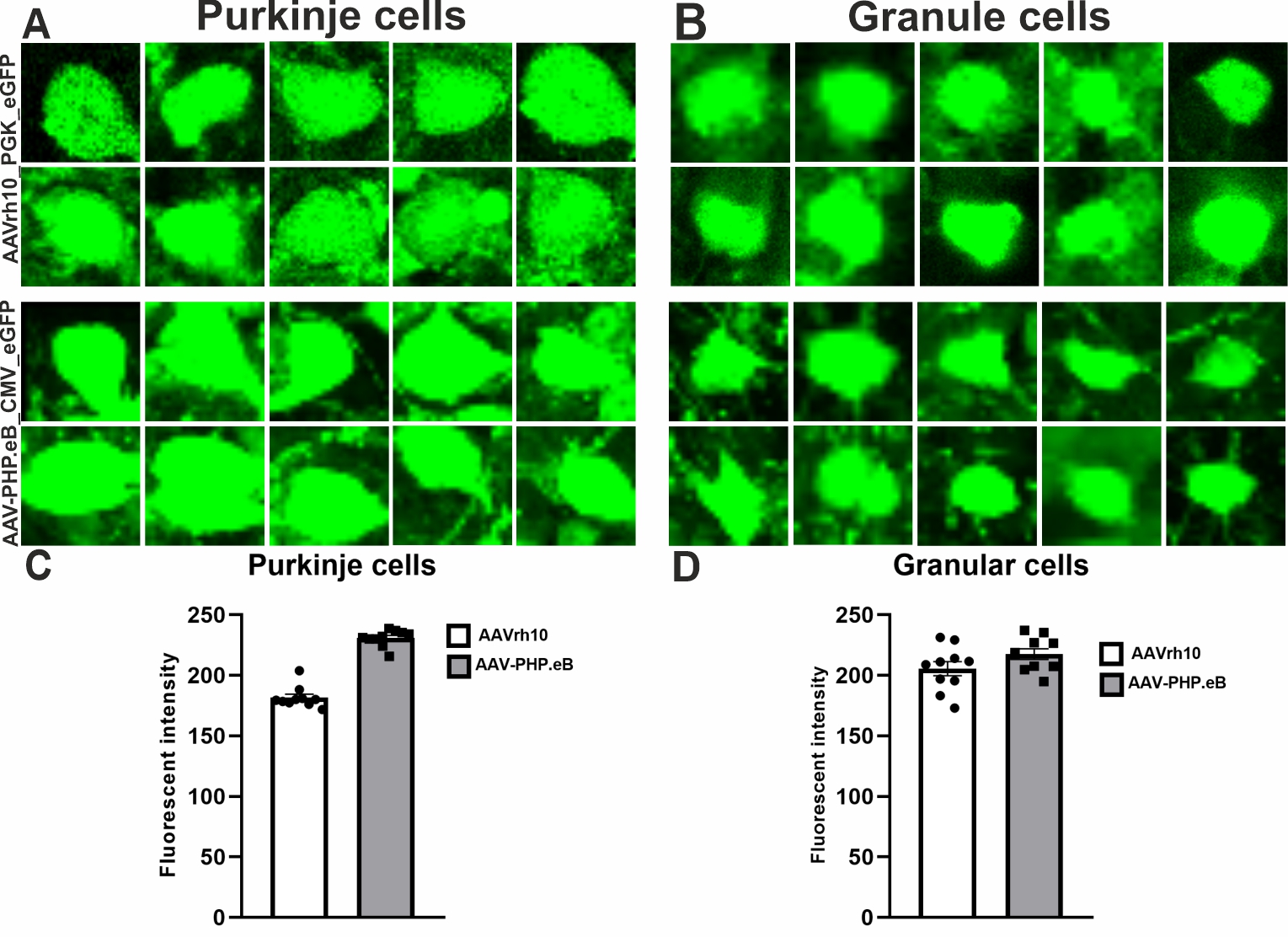

Supplement: Supplementary Figure S2 — Comparison of the activity of AAV cassettes containing eGFP under the control of CMV and PGK1 promoter in Purkinje and granule cells. Purkinje and granule cells were assayed by analyzing the fluorescent integrated optical density (IOD; Fiji/ImageJ) of the eGFP signal in single cells. Ten eGFP-positive cell bodies of Purkinje cells for each AAVrh10 (PGK promoter cassette) and AAV-PHP.eb (CMV promoter cassette) show a high level of eGFP by IOD measurement (A,C), and 10 cell bodies of granule cells for each cassette also show a high level of eGFP by IOD measurement (B,D). Both cassettes containing PGK and CMV promoters induce high levels of eGFP expression, and the measured IOD expression level is comparable in both PCs (C) and GCs (D). [file Image_2.JPEG]

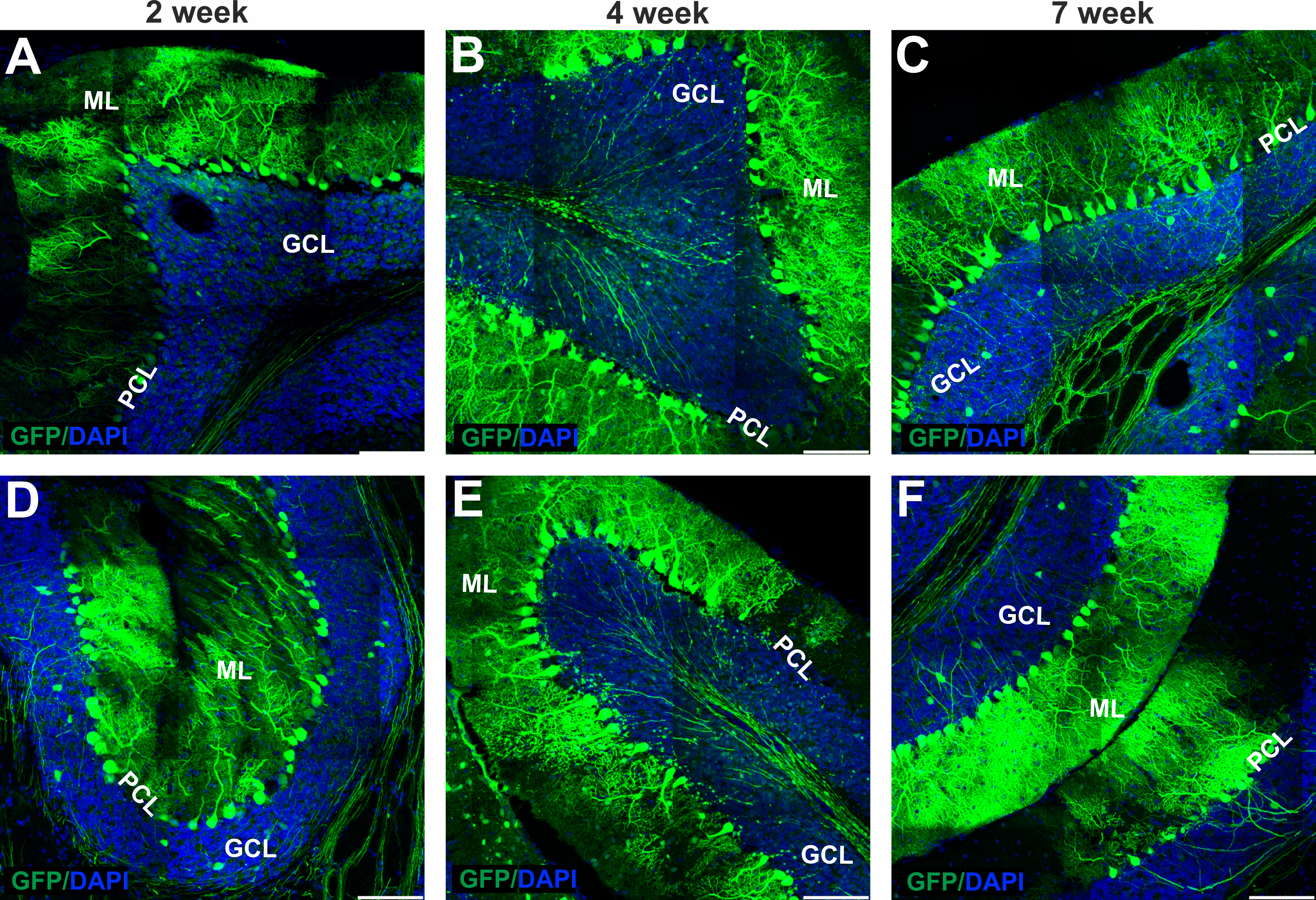

Supplement: Supplementary Figure S3 — Transduction pattern showing eGFP fluorescent signal after 2, 4, and 7 weeks post-DCN injection with AAV-PHP.eB. The figure demonstrates similar eGFP fluorescent intensity in Purkinje cells of two representative images for each time point: 2 (A,D), 4 (B,E), and 7 weeks (C,F) post-DCN injections. GCL, granule cell layer; ML, molecular layer; PCL, Purkinje cell layer; WM, white matter. Sagittal sections. Bars: 100 μm in the lower right corner of images. [file Image_3.JPEG]
